# Activation of immune defences against parasitoid wasps does not underlie the cost of infection

**DOI:** 10.3389/fimmu.2023.1275923

**Published:** 2023-12-07

**Authors:** Alexandre B. Leitão, Emma M. Geldman, Francis M. Jiggins

**Affiliations:** ^1^ Department of Genetics, University of Cambridge, Cambridge, United Kingdom; ^2^ Champalimaud Neuroscience Progamme, Champalimaud Centre for the Unknown, Champalimaud Foundation, Lisbon, Portugal

**Keywords:** *Drosophila*, parasitoid, immunity, cost, tradeoff

## Abstract

Parasites reduce the fitness of their hosts, and different causes of this damage have fundamentally different consequences for the evolution of immune defences. Damage to the host may result from the parasite directly harming its host, often due to the production of virulence factors that manipulate host physiology. Alternatively, the host may be harmed by the activation of its own immune defences, as these can be energetically demanding or cause self-harm. A well-studied model of the cost of infection is *Drosophila melanogaster* and its common natural enemy, parasitoid wasps. Infected *Drosophila* larvae rely on humoral and cellular immune mechanisms to form a capsule around the parasitoid egg and kill it. Infection results in a developmental delay and reduced adult body size. To disentangle the effects of virulence factors and immune defences on these costs, we artificially activated anti-parasitoid immune defences in the absence of virulence factors. Despite immune activation triggering extensive differentiation and proliferation of immune cells together with hyperglycaemia, it did not result in a developmental delay or reduced body size. We conclude that the costs of infection do not result from these aspects of the immune response and may instead result from the parasite directly damaging the host.

## Introduction

The reduction in host fitness during infection can be attributed to two major mechanisms. First, parasites may directly harm their hosts, frequently due to virulence factors that manipulate host physiology. For example, cholera toxin produced by *Vibrio cholerae* leads to an imbalance of electrolyte movement in enterocytes, which results in cellular water loss (1). Second, activation of immune defences can be costly. In insects, immune activation without infection can reduce fecundity [reviewed in (2)] and longevity (3). Immune responses can be costly for different reasons. Energy can be relocated from other physiological processes and development to the immune response (4). For example, when mice are injected with lipopolysaccharide, a potent immune stimulant, the energetic cost of the response causes them to enter a hypometabolic state accompanied by a decrease in body temperature (5). Immune responses can also cause self-harm (immunopathology), as exemplified by the activation of the melanisation cascade, an immune response in insects that damages self-tissues (6). Immune activation can also cause other physiological or behavioural changes, such as anorexia, that reduce fitness (7).

Because natural selection will favour traits that reduce the cost of infection, whether parasites harm their hosts directly or indirectly through a costly immune response has important evolutionary consequences. If the main cost of infection is the immune response itself, selection may favour a reduction in the magnitude of this response even if this increases susceptibility to infection (8). Such ‘immune restraint’ is unlikely if the direct harm caused by the parasite greatly outweighs the cost of mounting an immune response. Immune systems will also evolve adaptations to reduce costs, such as making immune responses inducible so that the cost of immunity is only incurred when infected (9, 10). For example, we recently demonstrated that aspects of the antiparasitoid immune response that are inducible when flies evolve under low parasitism conditions evolve to be constitutive when parasitism rates are high (11). This is predicted to be the case when immune responses are costly. Hence, to understand how immune defences evolve, we must understand why infection is costly.

When *Drosophila melanogaster* is infected with parasitoid wasps, larvae take longer to develop (12), and adults that survive the infection have reduced body size and fecundity (13). The immune response against parasitoids relies on both the humoral and cellular arms of the innate immune system. The humoral immune response includes upregulating complement-like proteins called TEPs (thioester-containing proteins) in the fat body (14, 15). The cellular immune response involves the proliferation and differentiation of different immune cell types. Large circulating immune cells called lamellocytes, which are rarely seen in healthy larvae, increase drastically in number after parasitoid infection (16). Together with plasmatocytes, they form a multi-layered capsule around the parasitoid egg to kill it. During the final stages of infection, lamellocytes activate prophenoloxidase 3 (PPO3), which is essential for the complete melanisation of the capsule around the parasitoid egg (17).

To manipulate the host immune response, parasitoid females frequently inject a cocktail of venoms into the larval haemocoel (18). In the genus *Leptopilina*, venom composition evolves very fast, allowing the diversification of strategies used by different wasp species. For example, the generalist species *L. heterotoma* uses protein with a hydrolase-like structure to lyse the lymph gland, the hematopoietic organ responsible for producing lamellocytes (19). A closely related species, *L. boulardi*, does not affect lymph gland structure, but it uses a RhoGAP protein to alter lamellocyte morphology, impairing the cell’s ability to encapsulate the wasp egg (20). Other proteins present in *L. boulardi* venom glands are predicted to affect glycolysis and other physiological processes (21).

Parasitoid infection is associated with major changes in metabolism, with infected larvae entering a state of hyperglycaemia triggered by the release of extracellular adenosine from immune cells (12). This metabolic switch is necessary to produce lamellocytes and the ability to encapsulate parasitoid eggs. It has been argued that the developmental delay and reduction in body size observed during parasitoid infection are caused by this metabolic shift and are therefore a cost of immune activation (12). However, parasite infection both exposes the host to virulence factors and activates the immune response, so either of these could be harming the fly. Here, we investigate whether reductions in body size and developmental delay are caused by the cost of mounting the encapsulation response.

## Results

### Injection of parasitoid wasp homogenate recapitulates the immune response to infection

To investigate the impact of immune activation on *D. melanogaster* physiology and life history, we compared larvae infected by the parasitoid wasp *L. boulardi* with larvae injected with ‘wasp homogenate’. Wasp homogenate is prepared by homogenising adult parasitoid wasps in paraffin oil, which is then injected into a *Drosophila* larva’s haemocoel. This induces the antiparasitoid immune response (22), with the oil droplet frequently being melanised similarly to wasp eggs [(22); **Supplementary Figures 1C, D**]. Virtually all fly larvae mount this response (22). However, because the homogenate is prepared from male wasps that do not have a venom gland, it does not include virulence factors. If paraffin oil is injected alone into larvae, it stays as a sphere and is not melanised [(22); **Supplementary Figure 1B**].

**Figure 1:**
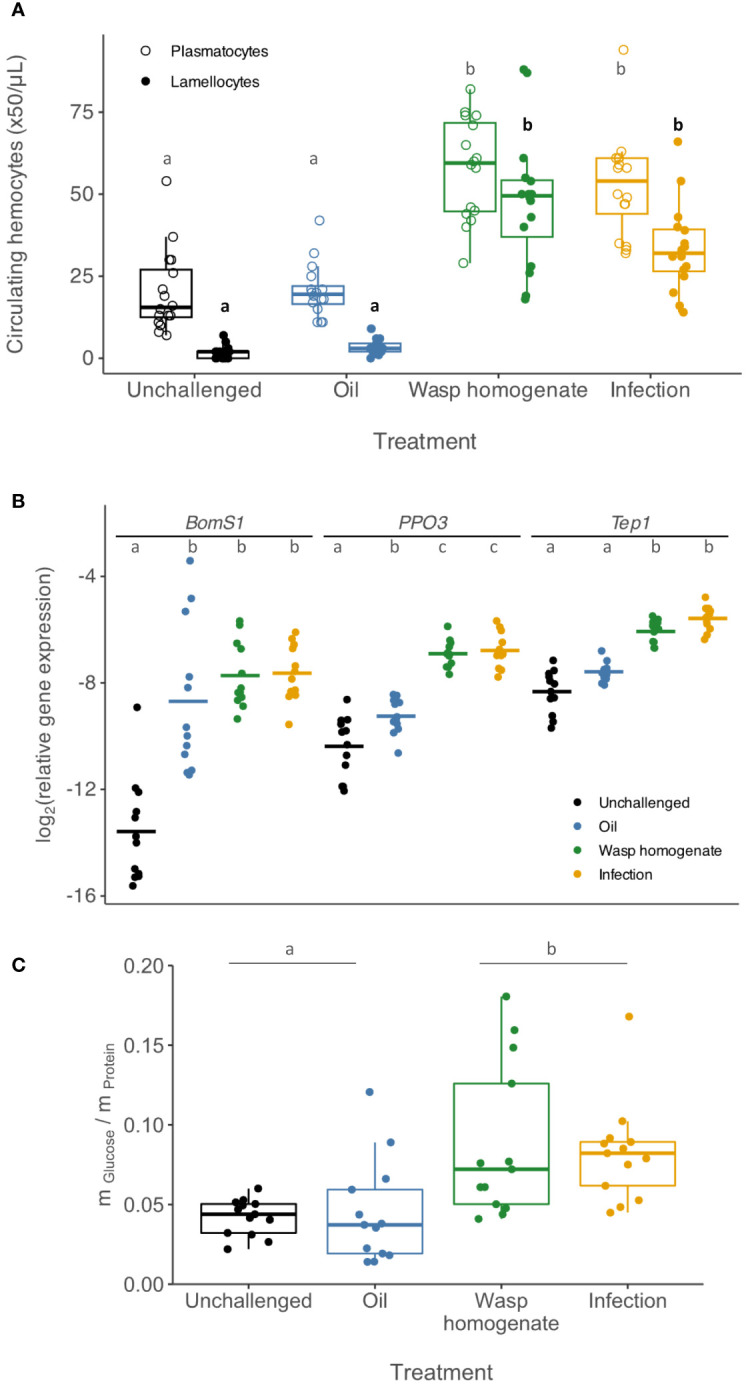
Effect of immune challenge on immunity and metabolism. Third instar larvae were injected with paraffin oil (blue), wasp homogenate (green) or parasitised (orange). **(A)** The concentrations of circulating plasmatocytes (open circles) and lamellocytes (dots) (16 pools of 8 – 10 larvae/treatment) 24h post-treatment. **(B)** Expression of three genes normalised to *RpL32* expression (n=11-12) 18h post-treatment. **(C)** The concentration of glucose relative to total protein was measured in cell-free hemolymph samples (13 pools of 40 larvae per treatment) 24h post-treatment. Boxes represent the 25^th^ and 75^th^ percentiles, and the line represents the median. In all cases, letters show significantly different groups (Tukey’s test, p <.05) In **(A)**, comparisons between lamellocytes are represented in bold.

Melanisation of the parasitoid egg occurs when it is surrounded by layers of plasmatocytes and lamellocytes that form a cellular capsule (16). When larvae were injected with wasp homogenate, the number of haemocytes in circulation at 24h increased to a level indistinguishable from that in infected larvae for both lamellocytes (**Figure 1A**, main effect of treatment: *χ^2 ^* = 298.75, d.f = 3, *p* < 2 x 10^-16^; wasp homogenate vs. infection: z = 2.15, *p* = 0.19) and plasmatocytes (main effect of treatment: *χ^2 ^* = 87.17, d.f = 3, *p* < 2 x 10^-16^; wasp homogenate vs. infection: z = 0.59, *p* = 1). In contrast, injection of oil caused a modest increase in lamellocytes (oil vs. unchallenged: difference in mean lamellocyte number = 1.85, z = 2.62, *p* = 0.05) and no increase in plasmatocytes (main effect of treatment: *χ^2 ^* = 87.17, d.f = 3, *p* < 2 x 10^-16^; oil vs. unchallenged: z = 0.26, *p* = 1). The melanisation response relies on the expression of *PPO3* in lamellocytes, and this gene was upregulated to a similar extent following the injection of wasp homogenate and infection (**Figure 1B**; main effect of treatment: *F* = 62.73, d.f = 3, *p* = 6.24 x 10^-16^; wasp homogenate vs. infection: t = 0.38, d.f. = 44, *p* = 1). Injection of oil alone caused a small increase in the expression of *PPO3* (oil vs. unchallenged: difference in Ct values = 1.13, *t* = 3.57, d.f. = 44, *p* = 0.005).

Alongside the cellular response, there is a humoral immune response to wasp infection that relies on the production of immune molecules by the fat body. This is characterised by the upregulation of the complement-like gene *thioester-containing protein 1* (*Tep1*), and this is induced to similar levels by both infection and wasp homogenate at 24h post-treatment (**Figure 1B**, main effect of treatment: *F* = 35.52, d.f = 3, *p* = 8.06 x 10^-12^; wasp homogenate vs. infection: t = 2.62, d.f. = 44, *p* = 0.07). Injection of oil alone caused only a small increase in *Tep1* expression (oil vs. unchallenged: difference in Ct values = 0.74, *t* = 2.55, d.f. = 44, *p* = 0.086). Wounding alone can induce a transcriptional change in the fat body (23), including genes involved in microbial killing, such as *Bomanin Short 1* (*BomS1*). Both wasp infection and injection result in a wound from the ovipositor or needle, and the induction of *BomS1* occurs to a similar level in all challenges (**Figure 1B**, main effect of treatment: *F* = 26.76, d.f = 3, *p* = 5.26 x 10^-10^; wasp homogenate vs. oil: *t* = 1.25, d.f. = 44, *p* = 1; infection vs. oil: *t* = 1.37, d.f. = 44, *p* = 1; infection vs. wasp homogenate: *t* = 0.118, d.f. = 44, *p* = 1).

Infection by parasitoid wasps causes a metabolic change characterised by a state of hyperglycaemia (12). Injection of wasp homogenate increased the concentration of circulating glucose to the same levels as infection at 18h (**Figure 1C**; main effect of treatment: *χ^2 ^* = 35.69, d.f = 3, *p* = 8.69 x 10^-8^; wasp homogenate vs. infection: z = 0.58, *p* = 1), while oil injection had similar levels to unchallenged larvae (oil vs. unchallenged: z = 0.223, *p* = 1). Overall, these results indicate that the injection of wasp homogenate induces similar physiological responses to parasitoid infection.

### Developmental delay and body size reduction results from infection but not experimental induction of the immune response

**Figure 2:**
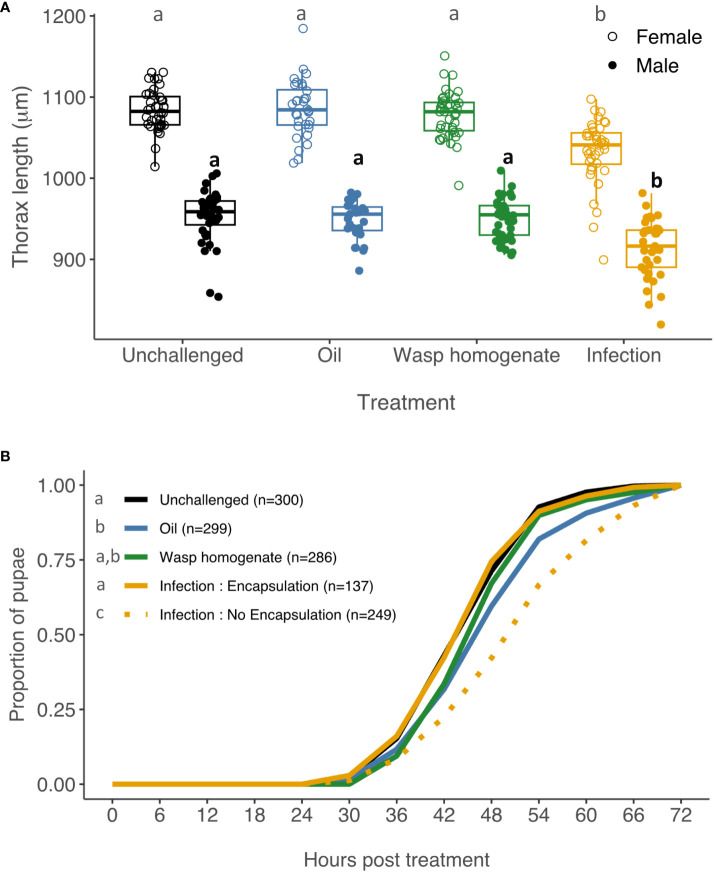
Effect of wasp infection and immune activation on body size and development time. Third instar larvae were injected with paraffin oil (blue), wasp homogenate (green) or parasitised by a female wasp (orange). **(A)** Thorax length was measured in females (open circles) and males (filled circles), *n*=27-40. **(B)** The number of larvae that pupated was recorded every 6 hours for 72 hours. Infected pupae were maintained to determine if the fly survived having successfully killed the parasitoid (Encapsulation, orange solid line) or if the wasp survived (No Encapsulation, orange dashed line). Number of tested larvae in parentheses. In all cases, letters show significantly different groups (Tukey’s test, p < 0.05). In **(A)**, comparisons between males are represented in bold.

As previously reported (13), infection by parasitoid wasps reduced thorax length by ~5% in females (**Figure 2A**, unchallenged vs. infection: *t* = 7.05, *p* = 3.9 x 10^-10^) and ~4% in males (unchallenged vs. infection: *t* = 5.77, *p* = 2.9 x 10^-7^). However, the thorax length of flies that were injected with wasp homogenate was indistinguishable from that of unchallenged larvae (females: *t* = 0.78, *p* = 1, males: *t* =0.34, *p* = 1) and larvae injected with oil (females: *t* = 0.25, *p* = 1, males: *t* = 0.32, *p* = 1). Therefore, the immune responses triggered by wasp homogenate cannot explain the reduction in body size following infection.

Infection by parasitoid wasps also causes a developmental delay (12). By the time they pupate, *Drosophila* that were infected as larvae have either mounted a successful immune response and encapsulated the parasitoid egg, or they have a living wasp larva feeding on their hemolymph. For this reason, when measuring the pupation timing of infected larvae, we distinguished between these two phenotypes. Interestingly, we observed that larvae that were successful in encapsulating the parasitoid egg did not have a developmental delay (**Figure 2B**, orange solid line, unchallenged vs. encapsulation: z = 0.04, *p* = 1). Larvae where the immune response failed and the parasite survived had a significant developmental delay of ~6 h (**Figure 2B**, orange dashed line, unchallenged vs. no encapsulation: z = 7.96, *p* = 1.78 x 10^-14^). This developmental delay was also highly significant when compared to larvae injected with either oil or oil and wasp homogenate (**Figure 2B**).

There was also a smaller cost to injecting oil, which may reflect harm caused by our injection protocol. The injection of oil caused a small developmental delay (**Figure 2B**; unchallenged vs. oil: z = 4.85, *p* = 1.25 x 10^-5^). The development time of the oil-injected flies did not differ significantly from larvae injected with oil containing wasp homogenate (wasp homogenate vs. oil: z = 2.35, *p* = 0.18).

## Discussion

Immune responses require drastic changes in gene expression and cell differentiation, and the energy necessary for these processes must be redirected from other traits. In addition, immune responses can damage the host’s own tissues. Both effects can result in trade-offs between immune competence and other fitness-related traits. However, to detect and quantify these trade-offs, it is necessary to have an artificial system to trigger the immune response, excluding the negative effects of virulence factors delivered by parasites. For example, the antibacterial immune response in mammals can be triggered by injecting LPS (5). In insects, the encapsulation response has been activated using materials such as nylon strings (24). In *Drosophila*, paraffin oil can activate the encapsulation response in some species (25), but this is less successful in *D. melanogaster* (26). Here, we exploited a technique we developed recently that involves injecting paraffin oil containing wasp homogenate (available as a preprint, 22). We found that the wasp homogenate in the oil triggers physiological changes that have been associated with costly immune responses, and the magnitude of these immune responses is similar to the response to natural infection by a parasitoid wasp. First, injection of wasp homogenate induces melanisation, which in other insect species is known to damage host tissues (6). Second, it induces an increase in the number of circulating hemocytes and the differentiation of lamellocytes. Finally, the injection of wasp homogenate causes flies to become hyperglycaemic. This is a signature of a metabolic switch previously reported with parasitoid infection (12), which is thought to underlie the reallocation of energy from development to the immune system. Importantly, our data demonstrate that this metabolic shift is triggered by immune challenge and is neither a manipulation by the parasitoid wasp nor an indirect effect of the wasp damaging host tissues.

The development of a technique to trigger the immune response allowed us to investigate whether the reduction in fitness suffered by flies that survive infection is a consequence of a costly immune response. The induction of the immune response is likely due to a pathogen associated molecular pattern (PAMP) present in adult wasps and not in the venom, since immune readouts are similar between wasp homogenate prepared with males or females. While the injection of oil droplets induces little transcriptional response in the fat body, injection of wasp homogenate induces a transcriptional response that resembles the one induced by parasitoid infection (22).

In agreement with past work, we found that infection by parasitoid wasps causes a reduction in body size (13). This is likely to reflect a reduction in fitness—for example, artificial selection experiments have found that smaller *D. melanogaster* males have lower fecundity and longevity (27). However, when we activated the immune response by injecting wasp homogenate, it did not affect body size. As we observed a reduction in body size in infected animals held under the same conditions, this suggests that this is not being caused by the immune responses triggered by wasp extract, which includes lamellocyte differentiation, a humoral response and hyperglycemia. Alternatively, smaller larvae could be more resistant to parasitoid infection, resulting in smaller adult in parasitized conditions. However, this scenario is unlikely since selection for *L. boulardi* resistance in *D. melanogaster* does not change body size over the generations (28).

This suggests that the cost of infection is caused by the parasite directly harming its host. As we are studying flies that succeed in killing the parasite, the parasite is normally killed as an egg before it can directly damage host tissues by feeding at the larval stage. Instead, the likely causes of reduced body size are the virulence factors injected by the female wasp at the time of infection or secreted by the developing wasp embryo. Similar reductions in body size have been observed with infections by another parasitoid species, *Asobara tabida* (13), which is very distantly related to *L. boulardi*. As these species have very different venom compositions (29), it is unclear whether there is a common mechanism for the reduction in body size caused by these different parasites. Combined with efforts to describe venom composition in parasitoid wasps (21, 30), it is now worth pursuing which venoms affect the fitness-related traits in the host.

An alternative explanation of why the wasp homogenate injection does not carry the same costs as infection is that it does not trigger the same immune response as a wasp egg. This was not the case for the traits we measured. Most notably, the wasp homogenate and infection induced similar levels of hyperglycaemia and lamellocyte differentiation, and these are the responses that have previously been thought to underlie the cost of infection (12). In a separate study we have used RNA sequencing (RNAseq) to investigate the effect of wasp homogenate on gene expression (22). In hemocytes, the treatment significantly altered the expression of 3886 genes, which allowed us to use our published single-cell RNAseq data to infer how the hemocyte population had changed (11, 22). This revealed a response to wasp homogenate that resembles the response to wasp infection (11). Not only did mature lamellocytes differentiate, but there are also increases in the proportion of immature lamellocytes—cells that morphologically resemble plasmatocytes and therefore could not be detected in the experiments reported here (11). We also used RNAseq and quantitative PCR (qPCR) to measure gene expression in the fat body, which is the primary organ in the humoral immune response (11). This confirmed that wasp homogenate upregulates Bomanins, which are a family of genes regulated by the Toll pathway (31). *Tep1* and *Tep2*, which are regulated by the JAK-STAT and Toll pathways (32), also increased in expression. Similarly, *IBIN*, which is regulated by the Toll and IMD pathways (33), was upregulated, as was *TotC*, which is regulated by the JAK-STAT pathway (34). Together these results indicate that the JAK-STAT and Toll pathways, which play a key role in the immune response to parasitoid wasps, are upregulated by wasp extract. Furthermore, upregulated genes are known to have important functions. Wasp extract increased the expression of both *Tep1* and *Lectin-24a* (35), which play a role in killing parasitoids, and *IBIN*, which affects blood sugar levels (33). However, it is possible that the duration of these responses or some other response that we did not measure may differ between natural infection and our experimental induction of the immune response. Moreover, it would be valuable to investigate whether the translation of upregulated immune genes is similar in both conditions, as transcription and translation do not always display a linear relationship during the insect immune response (36) and can underpin different costs.

Apart from the costs of deploying an immune response, that are manifested following infection, there can also be costs of maintenance of immune defences, that are manifested in the absence of infection. *D. melanogaster* populations selected to resist parasitoid infection have reduced competitive ability in low food conditions (37, 38), demonstrating that resistance to parasitoid wasps is costly to maintain. These populations show altered hemocyte composition in circulation, which is believed to be the cause for higher resistance and possibly for the cost of maintenance. It would be interesting to understand if immune activation with wasp homogenate induces costs in larva competitive ability with low food availability.

In line with past work, we found that flies that had been parasitised developed slower than uninfected controls. Again, we were unable to mimic this effect by upregulating the immune response. Here, the explanation was straightforward, as the developmental delay was found only in larvae that had failed to mount a successful immune response and contained a live parasite. This suggests that the developmental delay may be due to direct damage caused by the parasite. Developmental delay of the host can be beneficial for some parasitoid wasps (18). This could be the case for *L. boulardi*, for example, to guarantee that the host larva reaches an appropriate size. Alternatively, the developmental delay can be caused by an immune response that continued for a longer period in the larvae that failed to kill the parasite. Regardless, the developmental delay is of little evolutionary relevance for the host, as the flies will all be killed during the pupal stage by the parasite.

In an elegant study, Bajgar and colleagues showed that during parasitoid infection, nutrients are relocated from development into the differentiation of new immune cell types, particularly lamellocytes (12). This metabolic switch is dependent on sensing extracellular adenosine taken up by the adenosine receptor (AdoR). *AdoR* mutants produce fewer lamellocytes upon infection and, strikingly, show no developmental delay or reduction in body size (12). This result is surprising considering our data, as their results suggest that the reallocation of resources to the immune response underlies the costs of infection. One explanation of the apparent conflict between this result and our observations is that these costs result from an interaction between the effects of wasp infection and the host response, which is dependent on AdoR. For example, virulence factors may damage hosts only when there is an AdoR-mediated change to host metabolism. Alternatively, the genetic background of the *AdoR* mutants may have made them respond differently to infection. As fewer flies survive infection in the absence of the AdoR-mediated reallocation of resources to immunity, it is possible that those that do survive manage to kill the parasite larva immediately upon infection and are less seriously affected by parasite-secreted virulence factors.

Our results indicate that the large cost of infection seen in flies that survive parasitoid attack is not reproduced by experimental immune activation. Therefore, there may not be selection to moderate the magnitude of this aspect of the immune response if that reduces the probability of killing the parasite. This is particularly the case for this system, as failure to kill the parasite inevitably results in death. However, it is still possible that the activation of immune defences against parasitoid wasps may carry costs we were not able to measure. First, if we had measured the immune response at different times or other immune traits, we may have found a larger response in the presence of the wasp. This could be addressed by directly testing the fitness effects of exposure to virulence factors. Second, immune induction may affect fitness components we did not measure, or costs may only become apparent in certain environments. For example, our experiments were performed with food *ad libitum*, and some costs associated with immune defence are only visible with limited food resources (39).

## Methods

### *D. melanogaster* and *L. boulardi* maintenance

A wild-type *D. melanogaster* population was maintained with nonoverlapping generations with 200 flies per generation (11). Flies in this population encapsulate approximately 10% of the wasp strain used in our experiments (11). In each generation, flies were allowed to lay eggs overnight in 90 mm diameter agar plates (per 1500 ml water: 45 g agar, 50 g dextrose, 500 ml apple juice, 30 ml Nipagin 10% w/v) spread with a thin layer of yeast paste (Saccharomyces cerevisiae – Sigma−Aldrich #YSC2). Eggs were washed and removed with phosphate buffered saline (PBS) and a paintbrush, collected in 15 ml centrifuge tubes and allowed to settle on the bottom of the tube. Five hundred microliters of egg solution was transferred into a 1.5 ml microcentrifuge tube. Five microliters of egg suspension was transferred into plastic vials with cornmeal food (per 1200 ml water: 13 g agar, 105 g dextrose, 105 g maize, 23 g yeast, 35 ml Nipagin 10% w/v) and incubated at 25**°**C for 12-15 days in a 14-hr light/10-hr dark cycle and 70% humidity.

*Leptopilina boulardi* strain G486 (40) was maintained in a susceptible *D. melanogaster* outcrossed population named CAMOP2. Cornmeal vials were prepared with 6 μl of eggs as described above. Two female wasps and one male were added to each vial. Vials were incubated for 24 days at 25**°**C in a 14-hr light/10-hr dark cycle and 70% humidity. Adult wasps were collected and maintained in cornmeal vials with a drop of honey.

### Immune challenge treatments

To prepare wasp homogenate, 20 *L. boulardi* males were collected into a 0.5 mL microcentrifuge tube and homogenised in 200 μL of paraffin oil with a pestle. The wasp homogenate was centrifuged for 2 minutes at 300xg, and the supernatant was transferred into a new 0.5 mL microcentrifuge tube. Centrifugation was repeated, and the supernatant was transferred into a new 0.5 mL microcentrifuge tube.

Flies were allowed to lay eggs for four hours, and egg suspensions were prepared as described above. Fifteen microliters of eggs in PBS were then pipetted onto 50 mm diameter cornmeal plates and incubated at 25°C for 72 hours. Using forceps, 40 early 3^rd^ instar larvae per sample were gently collected and transferred onto filter paper. For unchallenged controls, larvae were directly transferred into a cornmeal vial by wetting the filter paper with double-distilled water (ddH_2_O) and collecting the larvae with forceps. For parasitism, the larvae were transferred into a cornmeal vial and exposed to three female G486-strain wasps for three hours. For injections, glass needles were prepared from 3.5” long borosilicate glass capillaries (Drummond Scientific Co. 3-000-203-G/X) pulled in a needle puller (Narishige PC-10). The needles were backfilled with paraffin oil or wasp homogenate, and 4.6 nL was injected into each of the 40 early third instar larvae that had been collected onto filter paper. The larvae were then transferred into a cornmeal vial. All vials were incubated at 25°C, 70% humidity and a 14-hour light/10-hour dark cycle.

### Hemocyte counts

A 15% w/v sugar solution was added to the vials to suspend the larvae, and 10-12 larvae were collected from each vial. The larvae were washed in double-distilled water (ddH2O), dried on filter paper and transferred into a cavity of a porcelain spot plate. The larvae were bled by tearing the cuticle of their ventral side, and 2 μL of hemolymph was diluted into 8 μL of Neutral Red staining solution (1.65 g/L PBS – Sigma−Aldrich #N2889). The stained hemolymph samples were loaded into hemocytometers (Thoma), and the numbers of plasmatocytes and lamellocytes in 0.1 μL were recorded. Hemocyte counts were performed at 24 hours post-treatment.

### Quantitative PCR

Larvae were transferred into cornmeal vials to be incubated in the constant temperature (CT) room for an additional 18 hours. The larvae were suspended in 15% w/v sugar solution, and 30 larvae from each vial were collected using forceps. The larvae were washed with ddH2O and dried on filter paper. Ten larvae from each vial were collected into 0.5 mL microcentrifuge tubes containing 1.0 mm diameter zirconia beads. Then, 250 μL of TRIzol reagent was added to each sample, and the samples were homogenised using a Qiagen Retsch MM300 TissueLyser set at a vibrational frequency of 25 Hz for 2 minutes. Samples were stored at -80°C.

For RNA extraction, samples were defrosted at room temperature and centrifuged for 10 minutes at 12,000xg and 4°C. Then, 160 μL of the supernatant was pipetted into a new 1.5 mL microcentrifuge tube, and 62.5 μL of chloroform was added to each tube. The tubes were shaken for 15 seconds and incubated at room temperature for 3 minutes. Following this, samples were then centrifuged for 10 minutes at 12,000xg and 4°C. A total of 66 μL of the aqueous phase was transferred into a new 1.5 mL microcentrifuge tube, and 156 μL of isopropanol was added to each sample. Tubes were inverted 5 times to mix the components thoroughly and incubated at room temperature for 10 minutes. Samples were then centrifuged for 10 minutes at 12,000xg and 4°C, and the supernatant of each sample was discarded. The pellets were washed with 250 μL of 70% ethanol and centrifuged for 2 minutes at 12,000xg and 4°C. The ethanol was discarded, and the pellet was dried. Then, 20 μL of nuclease-free water was added, and the samples were incubated at 45°C for 10 minutes. Samples were stored at -80°C.

For cDNA synthesis, samples were defrosted and kept on ice. cDNA was synthesised using the GoScript Reverse Transcription System Kit (Promega) following the manufacturer’s instructions. For quantitative real-time PCR (qPCR) on an Applied Biosystems StepOnePlus system, the SensiFASTTM Hi-Rox® SYBR kit (Bioline) was used. Primers used for the genes of interest: BomS1 (BomS1_Fw: 5’-ACCGGAGAAATCCATCCAGA-3’; BomS1_Rev: 5’-CGACAGTGGAACAGCATTGG-3’), Tep1 (Tep1_Fw: 5’-ACTGGAAGCCTCATTGGTCG-3’; Tep1_Rev: 5’-ACCGACAATGGGAACAGGAC-3’) and PPO3 (PPO3_Fw: 5’-GATGTGGACCGGCCTAACAA-3’; PPO3_Rev: 5’-GATGCCCTTAGCGTCATCCA-3’), and for *RpL32* (RpL32_Fw: 5’-TGCTAAGCTGTCGCACAAATGG-3’; RpL32_Rev: 5’-TGCGCTTGTTCGATCCGTAAC-3’) to normalise gene expression. A technical replicate was completed for each biological sample.

### Thorax measurements

Emerging adult fruit flies were collected and frozen at -20°C in 50 mm plates. The right wing of each fly was removed. Each fly was then positioned on a microscope slide, resting on their left side. Using a Leica Brightfield stereoscope mounted with a GXCapture camera, images of the thorax were taken, and measurements were recorded using ImageJ software. The thorax was measured as shown in **Supplementary Figure 2.**

### Circulating glucose quantification

The larvae were transferred into cornmeal vials and incubated in the CT room for an additional 24 hours. The larvae were suspended in 15% w/v sugar solution and collected using forceps. All 40 larvae from each vial were washed with ddH2O and dried on filter paper.

They were then transferred into a cavity of a porcelain spot plate and rapidly bled by tearing the cuticle of the ventral side. Eight microliters of hemolymph was collected and added to a 0.5 mL microcentrifuge tube containing 8 μL of PBS (pH 7.2). The samples were centrifuged for 5 minutes at 800xg and 4°C. Then, 4 μL of each sample was pipetted into a new 0.5 mL microcentrifuge tube to be used for protein quantification. Ten microliters of each sample was pipetted into another 0.5 mL microcentrifuge tube and heat-shocked at 75°C for 5 minutes. These were used for glucose quantification. If not for immediate use, samples were frozen at -80°C.

To measure the glucose concentration of each sample from a standard curve, 16 μL of 1 mg/mL glucose standard was diluted in 84 μL of PBS (pH 7.2) to a concentration of 0.16 mg/mL. From this, four 2-fold serial dilutions were made, resulting in standards ranging from 0.01 mg/mL to 0.16 mg/mL. Then, 30 μL of the glucose standards and a PBS blank were pipetted into the wells of a Falcon 96 Well U-Bottom Tissue Culture Treated plate. The hemolymph samples were centrifuged for 5 minutes at 10,000xg and 4°C. The samples were further diluted by adding 5 μL of each sample to 25 μL of PBS. Then, 30 μL of each diluted sample was pipetted into the same plate. The GO assay reagent was prepared from the Glucose (GO) Assay Kit (Sigma−Aldrich^®^) following the manufacturer’s instructions and maintained on ice. Then, 100 μL of the reagent was added to each well containing the glucose standards and the samples. The plate was covered with a lid and incubated at 37°C for 30 minutes. The reactions in the plate were stopped by adding 100 μL of 12 N H2SO4 to the wells. The plate was centrifuged in a swing bucket motor to remove bubbles. Using a SpectraMax id3 Multi-Mode Microplate Reader, the absorbance of each sample was measured at 540 nm. A standard curve for glucose concentrations was created using the absorbance readings of the standards, and the glucose concentrations of the undiluted hemolymph samples were calculated.

To measure the protein concentration of each sample from a standard curve, standards ranging from 0.2 mg/mL to 1.4 mg/mL bovine serum albumin (BSA), which increased in increments of 0.2 mg/mL, were made by diluting 2 mg/mL BSA standard in PBS (pH 7.2). Ten microliters of the BSA standards and a PBS blank were pipetted into the wells of a Falcon 96 Well U-Bottom Tissue Culture Treated plate. The hemolymph samples were centrifuged for 5 minutes at 10,000xg and 4°C. The samples were further diluted by adding 2 μL of each sample to 198 μL of PBS. Then, 10 μL of each diluted sample was pipetted into the same plate. Bradford reagent (200 μL) was added to each well containing the BSA standards and the samples. After 5 minutes, the absorbance of each sample was measured at 595 nm using a SpectraMax id3 Multi-Mode Microplate Reader. A standard curve for protein concentrations was created using the absorbance readings of the standards, and the protein concentrations of undiluted hemolymph samples were calculated. The circulating glucose in the hemolymph of 40 larvae was calculated by dividing the glucose concentration of each sample by the protein concentration.

## Data availability statement

The datasets presented in this study can be found in online repositories. The names of the repository/repositories and accession number(s) can be found below: https://www.repository.cam.ac.uk; https://doi.org/10.17863/CAM.104190.

## Ethics statement

The manuscript presents research on animals that do not require ethical approval for their study.

## Author contributions

AL: Conceptualization, Funding acquisition, Supervision, Writing – original draft, Data curation, Formal Analysis, Investigation, Methodology, Visualization. EG: Investigation, Writing – review & editing. FJ: Conceptualization, Funding acquisition, Supervision, Writing – original draft.

## Supplementary material

Supplementary Figure 1 Outcome of immune challenges. 3^rd^ instar larvae were injected with paraffin oil (blue), wasp homogenate (green) or parasitized by a female wasp (orange). 48h post treatment, oil droplets injected with wasp homogenate become melanized **(C´)**, while oil droplets injected alone remain un-melanized **(B´)**. Cuticle melanization of the wound caused by the needle can be observed (arrows in **(B)** and **(C)**). When infected by a wasp, a wasp larva **(D’)** or a melanized capsule **(D’’)** can be observed.

Supplementary Figure 2 Measurement of thorax length.
