# Supplementary material for: Activation of immune defences against parasitoid wasps does not underlie the cost of infection
Source: Front Immunol. 2023 Dec 7;14:1275923. doi: 10.3389/fimmu.2023.1275923 (PMC10733856; doi:10.3389/fimmu.2023.1275923)
Supplement: Supplementary Figure 1 — Outcome of immune challenges. 3rd instar larvae were injected with paraffin oil (blue), wasp homogenate (green) or parasitized by a female wasp (orange). 48h post treatment, oil droplets injected with wasp homogenate become melanized (C´), while oil droplets injected alone remain un-melanized (B´). Cuticle melanization of the wound caused by the needle can be observed (arrows in (B) and (C)). When infected by a wasp, a wasp larva (D’) or a melanized capsule (D’’) can be observed. [file Image_1.pdf]

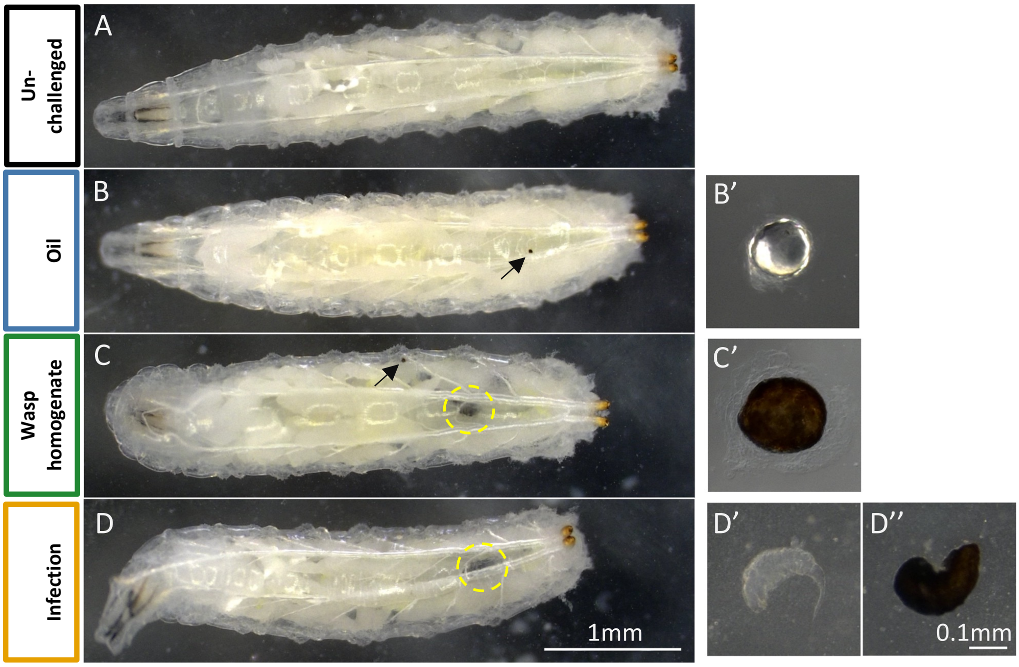

Supplementary Figure 1 – **Outcome of immune challenges** 3rd instar larvae were injected with paraffin oil (blue), wasp homogenate (green) or parasitized by a female wasp (orange). 48h post treatment, oil droplets injected with wasp homogenate become melanized (C´), while oil droplets injected alone remain un-melanized (B´). Cuticle melanization of the wound caused by the needle can be observed (arrows in (B) and (C)). When infected by a wasp, a wasp larva (D') or a melanized capsule (D'') can be observed.
