# Supplementary figures and images for: Activation of immune defences against parasitoid wasps does not underlie the cost of infection
Source: Front Immunol. 2023 Dec 7;14:1275923. doi: 10.3389/fimmu.2023.1275923 (PMC10733856; doi:10.3389/fimmu.2023.1275923)

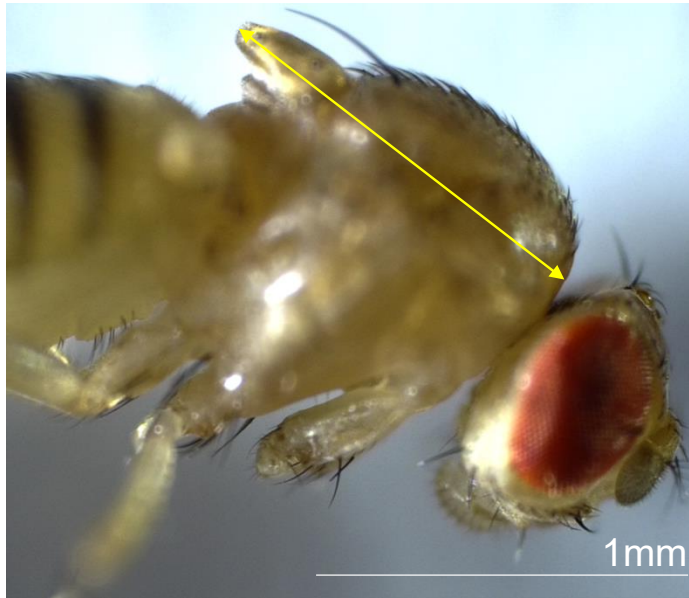

**Supplementary Figure 2. Measurement of thorax.**

Supplement: Supplementary Figure 2 — Measurement of thorax length. [file Image_2.pdf]
